# High Carbohydrate High Fat Diet Induced Hepatic Steatosis and Dyslipidemia Were Ameliorated by* Psidium guajava* Leaf Powder Supplementation in Rats

**DOI:** 10.1155/2019/1897237

**Published:** 2019-02-03

**Authors:** Md. Abdullah Al Mamun, Md. Faruk, Md. Mizanur Rahman, Kamrun Nahar, Fariha Kabir, Md Ashraful Alam, Nusrat Subhan

**Affiliations:** Department of Pharmaceutical Sciences, North South University, Bangladesh

## Abstract

*Psidium guajava* leaf is reported to contain many bioactive polyphenols which play an important role in the prevention and treatment of various diseases. Our investigation aimed to study the effect of* Psidium guajava* leaf powder supplementation on obesity and liver status by using experimental rats. To study the effects of guava leaf supplementation in high fat diet induced obesity, rats were randomly divided into four experimental groups (n=7), control (group I), control + guava leaf (group II), HCHF (group III), and HCHF + guava leaf (group IV). At the end of the experimental period (56 days), glucose intolerance, liver enzymes activities, antioxidant enzymes activities, and lipid and cholesterol profiles were evaluated. Our results revealed that guava leaf powder supplementation showed a significant reduction in fat deposition in obese rats. Moreover, liver enzyme functions were increased in high fat diet fed rats compared to the control rats significantly which were further ameliorated by guava leaf powder supplementation in high fat diet fed rats. High fat diet feeding also decreased the antioxidant enzyme functions and increased the lipid peroxidation products compared to the control rats. Guava leaf powder supplementation in high fat diet fed rats reduced the oxidative stress markers and reestablished antioxidant enzyme system in experimental animals. Guava leaf powder supplementation in high fat diet fed rats also showed a relative decrease in inflammatory cells infiltration and collagen deposition in the liver compared to the high fat diet fed rats. The present study suggests that the supplementation of guava leaf powder prevents obesity, improves glucose intolerance, and decreases inflammation and oxidative stress in liver of high carbohydrate high fat diet fed rats.

## 1. Introduction

Due to the changes in lifestyle and social and economic conditions, nowadays obesity is a global epidemic problem affecting both developing and developed nations [1, 2]. The prevalence of obesity is increasing throughout the world's population [3]. It is a chronic, multifactorial, and complex disease in which both genetic and environmental factors are involved [4]. It can reduce overall quality of life and lead to premature death. It may cause a wide array of health problems, such as diabetes, hypertension, cardiovascular disease, kidney disease, musculoskeletal disorders, sleep problems, and even some cancers [5]. Obesity is found to exacerbate some inflammatory pathways. Low-grade inflammation is associated with obesity which is an important mechanism to decrease insulin sensibility in adipose tissue, liver, and skeletal muscle [6]. Obesity causes excessive growth of adipose depots with adipocyte hypertrophy and hyperplasia [7]. This fat overload leads to an activation of inflammatory pathways and develops cellular insulin resistance [8]. Higher body weight increases the effort of movement, causing stress on the heart and muscles. Studies have showed a connection between intramyocellular lipid accumulation and reduced insulin mediated glucose uptake in skeletal muscle [9]. During insulin resistance in skeletal muscle, accumulation of intramyocellular lipid and inflammation impairs the insulin mediated glucose uptake in the skeletal muscle [10]. Glucose transport and glycogen synthesis are impaired, resulting in a reduced efficiency of glucose uptake and increased blood glucose delivered to the liver [11]. During hepatic insulin resistance, the increased lipid accumulation and inflammation impair the ability of insulin to inhibit gluconeogenesis and this leads to an increased glucose output [12]. Thus, lipogenesis remains unaffected and, with the increased supply of dietary glucose, leads to increased lipogenesis and may cause nonalcoholic fatty liver disease [13]. Obese persons often have increased levels of free fatty acids (FFA) together with the hyperglycemia [14]. FFA has shown to increase insulin secretion and when high FFA levels are chronically high, this phenomenon has shown to impair glucose-stimulated insulin secretion [15]. Fatty acids are also reported to induce apoptosis of *β*-cells* in vitro*, by a mechanism called lipotoxicity [16]. Obesity may also lead to steatosis and steatohepatitis, and fatty liver is vulnerable to oxidative stress [17].

Plants have been used traditionally for healing many diseases [18]. In particular, various oriental medicinal plants are reported to be efficacious in treating various diseases.* Psidium guajava *is one of the valuable parts in folk medicine and is believed to be pharmacologically active [19]. The ethnobotanical studies and folklore claims reviewed that the leaves of the guava were used for antioxidant, hepatoprotective, antiallergy, antimicrobial, antigenotoxic, antiplasmodial, cytotoxic, antispasmodic, cardioactive, anticough, antidiabetic, anti-inflammatory, and antinociceptive activities [20]. The tea made up from guava leaves is commonly used against gastroenteritis (dysentry) and child diarrhea [21]. Guava leaves mostly contain essential oils, tannins, flavonoids, phenol compounds, carotenoids, and vitamin C [22]. The budding leaves of* Psidium guajava* contained large amounts of soluble polyphenolics including gallic acid, catechin, epicatechin, quercetin, and rutin [23]. A mixture of sesquiterpene hydrocarbons (54.9%) and oxygenated sesquiterpenes (20.9%) with *β*-caryophyllene (18.3%) as the principal sesquiterpene hydrocarbon and selin-11-en-4-*α*-ol (6.9%), *α*-cadinol (3.6%), and (E)-nerolidol (3.2%) as the main oxygenated sesquiterpenes are reported to be present in the oil of* Psidium guajava* leaves [24]. Considering all the above factors and problems related to the obesity, here we tried to study the effects of guava leaves on high carbohydrate high fat (HCHF) diet induced obesity and the related pathophysiological complications in wistar rats.

## 2. Materials and Methods

### 2.1. Plant Materials

Guava leaves were collected from local area of Dhaka city, Bangladesh. Leaves were dried in Phytochemistry Laboratory at Department of Pharmaceutical Sciences. Dried leaves were then grinded to fine powder in a grinding machine. This fine powder was used as a supplement to the food used in this study.

### 2.2. Experimental Animals and Groups

Wistar male rats (28, ten to twelve weeks old, 185-200 g) were obtained from Animal Production Unit of Animal House at Department of Pharmaceutical Sciences, North South University. All rats were kept in individual cages at temperature controlled room (22±3°C, humidity 55% with a 12 h dark/light cycles). These rats were given free access to standard laboratory feed and water. Ethical Committee of North South University for animal care and experimentation approved the experimental protocols (AEC-003-2015). To study the effects of guava leves supplementation in high carbohydrate high fat diet (HCHF) fed animals, all rats were randomly divided into four experimental groups (n=7), control (group I), control + guava leaf (group II), HCHF (group III), and HCHF + guava leaf (group IV). Animals of group I were given the normal laboratory food and water up to the end of the study (8 weeks). Group II received a treatment similar to that of group I as well as guava leaf powder every day for 8 weeks. Rats of group III received only HCHF diet whereas animals of group IV received guava leaves mixed in HCHF diet every day (2.5% of powder food, w/w) for 8 weeks. The pellet forms of HCHF diet were prepared in our laboratory, using the previously reported formula from our lab (Table 1) [25, 26]. OGTT test was performed for all four groups of rats before and after finishing the experimental period to assess the glycemic activity. Body weight and food and water intake measurements were recorded daily. After 56 days, all animals were weighed and sacrificed by high dose of pentobarbital anesthesia (90 mg/Kg). The blood was collected into heparinized tubes and all internal organs like heart, kidney, spleen, and liver were also hervested. All the organs immediately after collection were weighed and stored in neutral buffered formalin (pH 7.4) for histological analysis and in refrigerator at −20°C for further studies. Blood was centrifuged at 8000 rpm (at 4°C for 15 min) within 30 min of collection to separate the plasma. Separated plasma was transferred to eppendorf tubes to store at −20°C for further analysis.

### 2.3. Oral Glucose Tolerance Test

Rats were kept starved overnight (12 h) and provided with normal water only. The next day morning, an oral glucose tolerance test (OGTT) was performed on these starved rats. Tail vein blood from rats was taken to measure basal blood glucose concentrations using a glucometer (Bionim Corporation, Bedford, MA, USA). After that, all rats were administered 2 g/kg body weight of glucose as a 40% aqueous solution via oral gavage. Tail vein blood samples were again taken at 30, 60, 90, and 120 min following glucose administration and analyzed the blood glucose concentration by a glucometer.

### 2.4. Assessment of Liver Function Markers

Liver function marker enzymes such as aspartate aminotransferase (AST), alanine aminotransferase (ALT), and alkaline phosphatase (ALP) activities were estimated in plasma by using DCI diagnostics kits (Budapest, Hungary) following the manufacturer's standard protocol.

### 2.5. Assessment of Cholesterol and Triglyceride

Cholesterol and triglyceride were measured in plasma by using the cholesterol and triglyceride assessment kits of DCI diagnostics (Budapest, Hungary) and the manufacturer's prescribed protocol was followed.

### 2.6. Assessment of Oxidative Stress Markers in Plasma and Tissues

Liver tissue (1 gm) was homogenized in 10 mL of phosphate buffer (pH 7.4) using a tissue homogenizer. The mixture was centrifuged at 8000 rpm for 15 min at 4°C. The supernatant was collected and stored at −20°C until further analysis. These samples were used for the determination of proteins and biochemical analysis as described below. Thiobarbituric acid reactive substances (TBARS) in plasma and tissue samples are the index of lipid peroxidation and oxidative stress. Lipid peroxidation in liver was estimated calorimetrically by measuring thiobarbituric acid reactive substances (TBARS) followed by the previously described method [27].

Assay of nitric oxide (NO) was conducted according to the method described by Tracy et al. as nitrate and nitrite [28]. NO level was measured by using standard curve and expressed as mmol/gm of tissue.

Determination of advanced protein oxidation product (APOP) concentration in the plasma and tissue samples was performed by the method described previously by Witko-Sarsat [29] and [30]. APOP concentrations were expressed as *μ*mol·L^−1^ chloramine-T equivalents.

### 2.7. Estimation of Catalase Activity (CAT), Superoxide Dismutase (SOD) Activity, and GSH Concentration

Catalase activities were determined by the method of Chance and Maehly [31] with some modifications. One unit of CAT activity was defined as an absorbance change of 0.01 as units/min.

The SOD activities were assayed in plasma and tissue homogenates by using previously described method [32]. One unit of enzyme activity has been defined to cause 50% inhibition of autooxidation of epinephrine present in the assay system.

Reduced glutathione (GSH) concentration was estimated followed by the previously published method of Jollow et al. [33].

### 2.8. Estimation of MPO Activity in Liver Tissue

The MPO activity in liver tissue was determined by a dianisidine-H_2_O_2_ method reported previously [34]. The change in absorbance was measured at 460 nm. Results were expressed as units of MPO/mg protein.

### 2.9. Histopathological Determination

Microscopic evaluation of liver tissues was performed to find the abnormalities in liver due to high fat diet feeding in rats. Liver tissues were fixed in neutral buffered formalin and embedded in paraffin wax to prepare the mould. The moulded tissues were sectioned at 5 *μ*m. These thin sections were stained with hematoxylin-eosin to see the hepatic tissue orientation and organization, fat deposition, and inflammatory cell infiltration. Sirius red staining was done for detecting fibrosis in liver sections. Prussian blue staining was also done to see the ferric ion deposition in liver tissues. Sections were then studied and photographed under light microscope (Zeiss Axioscope) at 40 X magnifications.

### 2.10. Statistical Analysis

All values are expressed as mean ± standard error of the mean (SEM). The results were evaluated by using the two-way or one-way ANOVA followed by Newman-Keuls multiple comparisons test using Graph Pad Prism Software, version 6. Statistical significance was considered at* p* < 0.05 in all cases.

## 3. Results

*Food and Water Intake*. Daily food and water intake were measured for each rat for 8 weeks. Control rats consumed 17.81±0.94 g food and 24.0±2.05 mL water every day, whereas HCHF diet fed rats consumed 15.03±0.96 g food and 20.79±2.17 mL water every day. Guava leaves supplemented rats did not alter the food (16.2±1.4 g) consumption but the water consumption was reduced (17.9±1.9 mL water per day) in high fat diet fed rats. Guava leaves supplementation in control rats also showed no change in food consumption (18.1±2.7 g) compared to the control rats only, whereas water consumption reduced to 18.3±1.9 mL.

### 3.1. Effect of Guava Leaf Powder Supplementation on Oral Glucose Tolerance Test (OGTT)

**Figure 1:**
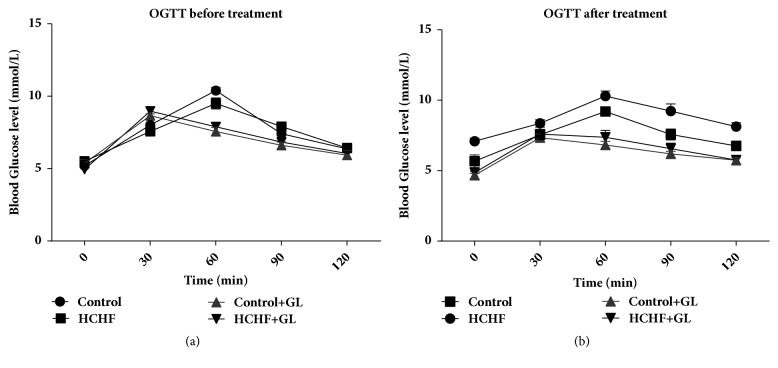
Effect of guava leaf powder supplementation on oral glucose tolerance test (OGTT) before and after the treatment in high fat diet induced obese rats. Values are presented as mean±SEM, n=7. One-way ANOVA followed by Newman-Keul's multiple comparisons test was done for statistical comparison. Values are considered significant at* p*<0.05.

Oral glucose tolerance test (OGTT) data are presented in Figure 1.  Figure 1(a) showed that all rats in all groups before entering into the high fat diet feeding protocol were capable of metabolizing the given glucose load and lowered the plasma glucose level to basal level. However, at the end of high fat diet feeding protocol, control rats lowered elevation in blood glucose level after glucose load and it declined to near basal level at 120 min, whereas, in HCHF diet-induced obese rats, the blood glucose level was noticed to be high even after 60 min and remained high over the next 60 min. Interestingly, supplementation of guava leaf powder to HCHF diet induced obese rats showed a significant decrease in blood glucose level at 60 min and beyond when compared with HCHF control rats (Figure 1(b)).

### 3.2. Effect of Guava Leaf Powder Supplementation on Liver Wet Weight

**Figure 2:**
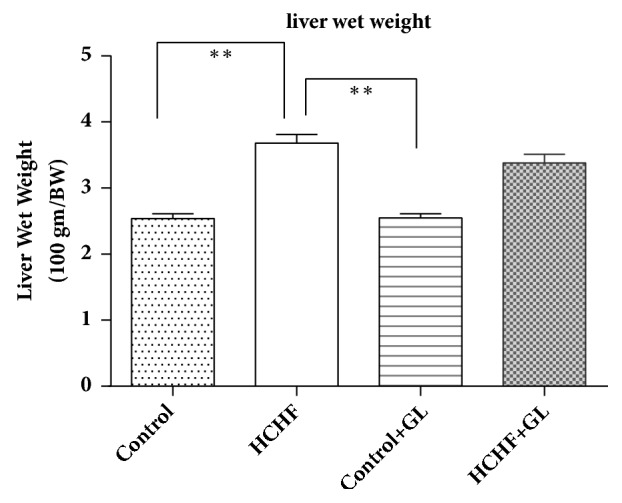
Effect of guava leaf powder supplementation on liver wet weights in high fat diet induced obese rats. One-way ANOVA followed by Newman-Keul's multiple comparisons test was done for statistical comparison. Values are considered significant at* p*<0.05.

The liver wet weights are presented in Figure 2. The liver wet weight was significantly (*p* < 0.05) increased in the HCHF diet fed rats in comparison to the control rats. Guava leaf powder (2.5% w/w of diet) supplementation significantly (*p* < 0.05) attenuated the increased wet weight of the liver in the HCHF diet fed rats. Moreover, normal rats treated with the guava leaves supplementation also showed a significant decrease in liver wet weight compared to control rats (Figure 2).

### 3.3. Effect of Guava Leaf Powder Supplementation on Accumulation of Fat in Adipose Tissues

**Figure 3:**
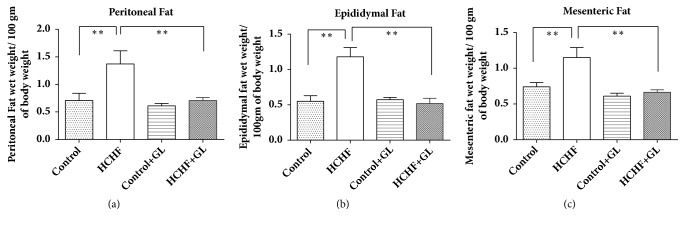
Effect of guava leaf powder supplementation on adipose tissue wet weights in high fat diet induced obese rats. One-way ANOVA followed by Newman-Keul's multiple comparisons test was done for statistical comparison. Values are considered significant at* p*<0.05.

Three types of fat accumulation have been presented in Figure 3. Vivid differences in fat deposition levels were observed in terms of retroperitoneal, mesenteric, and epididymal tissues, amongst the groups studied. The weights of retroperitoneal, mesenteric, and epididymal adipose fat tissues were markedly increased in HCHF diet fed rats compared to the control rats (Figure 3). Guava leaf powder supplementation reduced the weight of peritoneal and** e**pididymal fat tissues considerably compared to HCHF diet fed rats.

### 3.4. Effect of Guava Leaf Powder Supplementation on Liver Function Markers Enzyme Activities such as AST, ALP, and ALT

**Figure 4:**
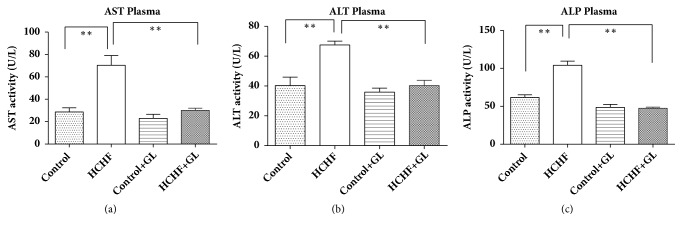
Effect of guava leaf powder supplementation on liver enzymes activities in plasma in high fat diet induced obese rats. Values are presented as mean±SEM, n=7. One-way ANOVA followed by Newman-Keul's multiple comparisons test was done for statistical comparison. Values are considered significant at* p*<0.05.

Liver function marker enzymes activities such as AST, ALT, and ALP results are presented in Figure 4. AST activities were increased significantly (*p*<0.05) in plasma of HCHF diet fed rats compared to the control rats which were normalized by guava leaves powder supplementation (Figure 4(a)). The ALT activities were also increased in plasma of HCHF diet fed rats compared to the control group of rats where the treatment with guava leaf significantly normalized ALT activities (Figure 4(b)). The ALP activities were also increased in plasma of HCHF diet fed rats compared to the control rats. Guava leaves powder supplementation significantly normalized the ALP activities (Figure 4(c)). Moreover, guava leaves powder supplementation did not alter any of the enzymes activities in plasma of control rats.

### 3.5. Effect of Guava Leaf Powder Supplementation on Cholesterol and Triglyceride Level

**Figure 5:**
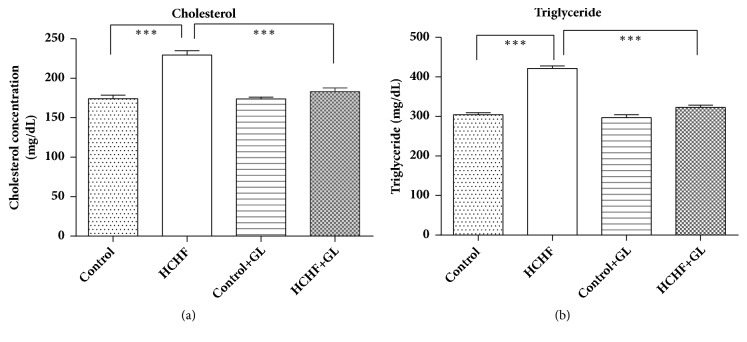
Effect of guava leaf powder supplementation on cholesterol and triglyceride level in plasma in high fat diet induced obese rats. Values are presented as mean±SEM, n=7. One-way ANOVA followed by Newman-Keul's multiple comparisons test was done for statistical comparison. Values are considered significant at* p*<0.05.

Lipid parameters are presented in Figure 5. HCHF diet fed rats showed increased level of total cholesterol compared to the control rats (Figure 5(a)). Treatment with guava leaf supplementation showed a significant reduction of cholesterol level in comparison to the HCHF diet induced obese rats. HCHF diet fed rats showed increased level of triglyceride compared to the control rats (Figure 5(b)). Treatment with guava leaf significantly lowered triglyceride level in plasma of HCHF diet induced obese rats (Figure 5(b)).

### 3.6. Effect of Guava Leaf Powder Supplementation on Oxidative Stress Markers and Antioxidant Enzymes

**Figure 6:**
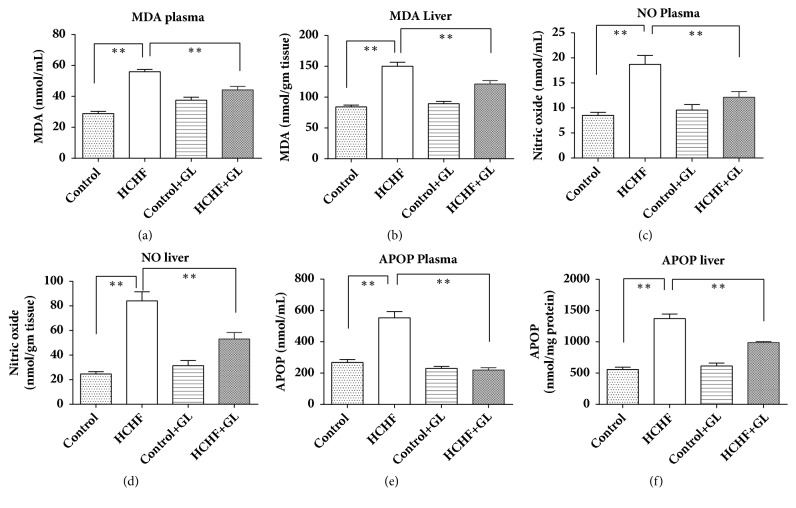
Effect of guava leaf powder supplementation on oxidative stress parameters in plasma and tissues of high fat diet induced obese rats. Values are presented as mean±SEM, n=7. One-way ANOVA followed by Newman-Keul's multiple comparisons test was done for statistical comparison. Values are considered significant at* p*<0.05.

To assess the oxidative stress condition of plasma and liver in high fat diet fed rats, MDA, NO, and APOP concentrations were measured. Lipid peroxidation was evaluated as MDA formation and is presented in Figure 6. MDA concentration in plasma and liver was significantly raised in HCHF diet fed rats compared to the control rats. On the contrary, guava leaf treatment in high fat diet fed rats prevented the rise of MDA concentration compared to HCHF diet fed rats. Another oxidative stress marker NO was significantly increased in plasma and liver of HCHF diet fed rats and was decreased in guava leaf supplemented rats (Figure 6). APOP concentration was also elevated in HCHF diet fed rats compared to control group rats. Treatment with guava leaf exhibited significant decreases in APOP concentration in both plasma and liver (Figure 6).

### 3.7. Effect of Guava Leaf Powder Supplementation on Antioxidant Enzymes Status (Catalase, SOD, and GSH)

**Figure 7:**
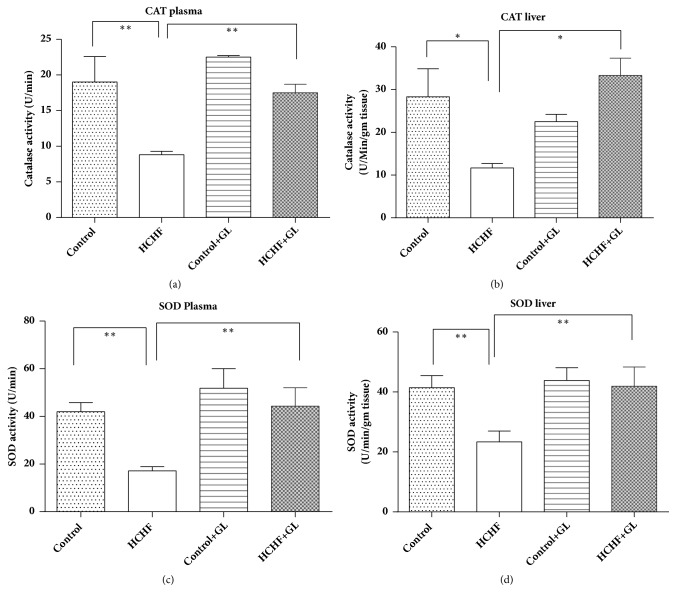
Effect of guava leaf powder supplementation on antioxidant enzyme activities in plasma and tissues of high fat diet induced obese rats. Values are presented as mean±SEM, n=7. One-way ANOVA followed by Newman-Keul's multiple comparisons test was done for statistical comparison. Values are considered significant at* p*<0.05.

Antioxidant enzymes catalase and SOD prevent the oxidative stress condition in plasma and liver. Activity of catalase was significantly depleted in HCHF rats compared to control whereas catalase activity was significantly increased in rats treated with guava leaf powder in both plasma and liver (Figure 7(a)). SOD activities were also significantly depleted in HCHF diet fed rats compared to the control rats (Figure 7(b)). Guava leaf treatment in rats restored the level of SOD activities in plasma and liver of HCHF diet fed rats (Figure 7(b)).

**Figure 8:**
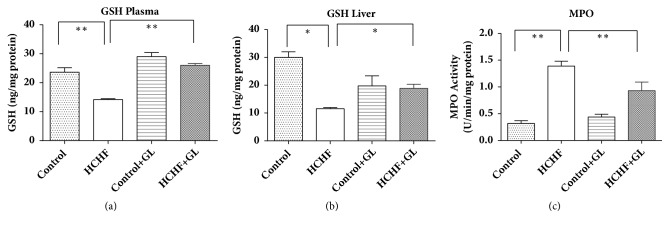
Effect of guava leaf powder supplementation on antioxidant GSH level in plasma and tissues and MPO activity in liver tissue of high fat diet induced obese rats. Values are presented as mean±SEM, n=7. One-way ANOVA followed by Newman-Keul's multiple comparisons test was done for statistical comparison. Values are considered significant at* p*<0.05.

GSH concentration was also depleted in plasma and liver tissue of HCHF diet fed animal compared to the control rats (Figure 8). Guava leaves supplementation restored the reduced GSH level in HCHF diet fed animals in both plasma and liver tissues (Figure 8).

### 3.8. Effect of Guava Leaf Powder Supplementation on Myeloperoxidase (MPO) Activity in Liver Tissue.

MPO activity was also increased in liver tissue of HCHF diet fed animal compared to the control rats (Figure 8). Guava leaves supplementation prevented the rise of MPO activity in liver tissues of HCHF diet fed animals (Figure 8).

### 3.9. Effect of Guava Leaf Powder on Histological Changes in Liver of HCHF Diet Rats

**Figure 9:**
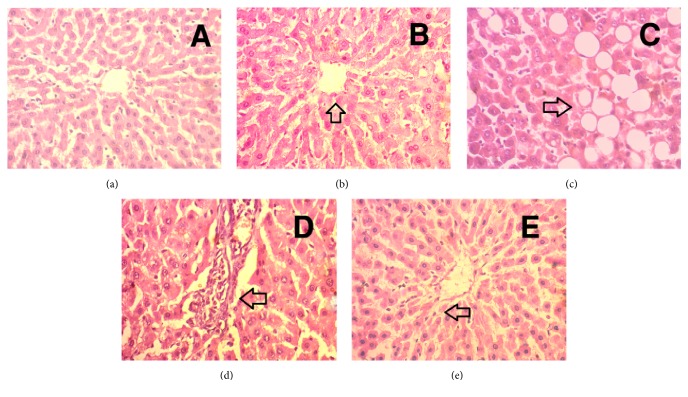
Effect of guava leaf powder on hepatic inflammatory cells infiltration in HCHF diet rats. (a) Control, arrowhead showed normal hepatic cells and bile duct structure; (b) control+ guava leaves, arrowhead showed similar structure like in control rats with normal histoarchitecture; (c) HCHF, arrowhead showed fat droplet deposition; (d) HCHF, arrowhead showed inflammatory cells infiltration around blood vessel and bile duct region in liver; (e) HCHF+ guava leaves, arrowhead showed no fat droplet deposition and it was devoid of any inflammatory cells infiltration. Magnification: 40 x 10 = 400 times.

*Hematoxylin and Eosin Staining (H & E Staining)*. The liver of control group showed comparatively intact and homogenous histoarchitecture with no necrosis and inflammation (Figure 9(a)). The liver of control group treated with guava leaf also showed intact and homogenous histoarchitecture with no necrosis and inflammation (Figure 9(b)). Liver of HCHF diet fed rats showed pivotal fat deposits with inflammatory cell infiltration compared to control group rats (Figures 9(c) and 9(d)). The HCHF diet fed rats treated with guava leaf showed protection from hepatic injury evidenced by decreased necrosis as well as inflammatory cell infiltration compared to HCHF group (Figure 9(e)).

**Figure 10:**
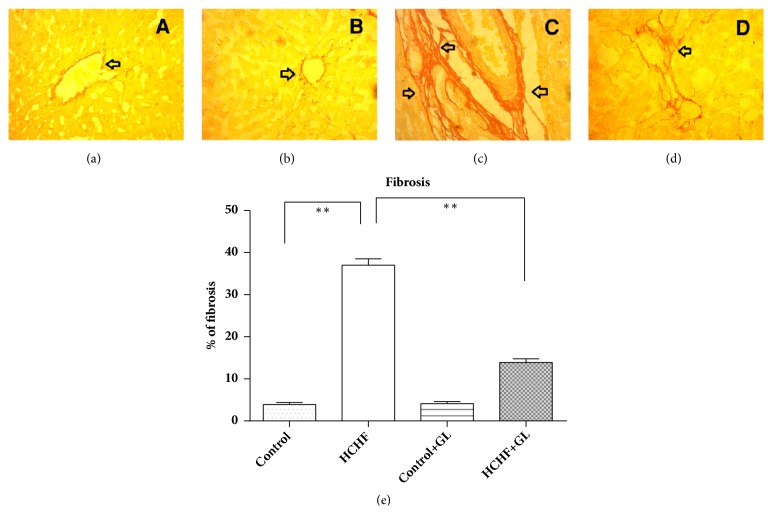
Effect of guava leaf powder on hepatic fibrosis in HCHF diet rats. (a) Control, arrowhead showed baseline collagen fiber around the bile duct; (b) control + guava leaves, similar to control, arrowhead showed baseline collagen fiber around the bile duct; (c) HCHF, arrowhead showed heavy collagen fiber was deposited around the blood vessel and bile duct; (d) HCHF+ guava leaves, arrowhead showed reduced collagen fiber deposition around the blood vessel and bile duct; (e) the % of fibrosis development in various liver tissues. Magnification: 10 x 40 = 400 times.

*Picrosirius Red Staining*. The image of control rats nearly showed normal collagen distribution and alignments in liver (Figure 10(a)). The liver of control group treated with guava leaf also showed normal collagen distribution and alignments (Figure 10(b)). HCHF diet fed rats showed excess collagen deposition and fibrosis compared to the control rats (Figure 10(c)). Guava leaf treatment significantly prevented the collagen deposition and fibrosis in HCHF diet fed rats (Figure 10(d)). The percentages of fibrosis were analyzed by using Image J free software from National Institute of Health and are presented in Figure 10(e). HCHF diet fed rats showed significantly increased fibrosis percentage compared to control rats, which was reduced significantly by guava leaves powder supplementation in HCHF diet fed rats (Figure 10(e)).

**Figure 11:**
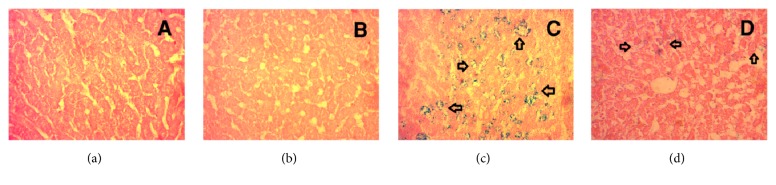
Effect of guava leaf powder on hepatic ferric ion deposition in HCHF diet rats. (a) Control, no blue droplet for iron was observed in liver tissue; (b) control + guava leaves, similar to control, no blue droplet for iron deposition was observed in liver tissue; (c) HCHF, arrowhead showed blue droplet for iron deposition in liver tissue; (d) HCHF + guava leaves, arrowhead showed reduced zone of blue droplet for iron deposition in liver tissue. Magnification: 10 x 40 = 400 times.

*Prussian Blue*. The control rats showed no ferric ion deposition in liver (Figure 11(a)). The liver of control group treated with guava leaf powder also showed no deposition of ferric ion (Figure 11(b)). HCHF diet fed rats showed excess ferric ion deposition compared to the control rats (Figure 11(c)). Guava leaf treatment significantly prevented the ferric ion deposition in HCHF diet fed rats (Figure 11(d)).

## 4. Discussion

Obesity is a major causative factor for the development of metabolic syndrome [35]. In obesity, amount of white adipose tissue in the body is increased [36]. High fat diet induced obesity in animal has been considered as the most popular model among researchers, because high fat diet produces high similarity of mimicking the usual route of obesity episodes in human [37, 38]. In this study, guava leaf powder supplementation is used to prevent obesity related metabolic syndrome in HCHF diet fed animals.

Guava leaf contains high amount of phenolic and flavonoid components which have important biological activity [22]. This investigation suggests that guava leaves supplementation prevented the glucose intolerance in high fat diet fed animals. Previous report also suggests that the decoction of the leaves was tested for hypoglycemic activity in alloxan induced diabetic rats and the extract showed a significant hypoglycemic activity [39]. Another study also showed that* Psidium guajava* aqueous leaf extract prevented LDL glycation in a dose-dependent manner [40]. The observed hypoglycemic activity by* Psidium guajava* could be attributed to the chemical compounds present in the extract such as tannins, flavonoids, pentacyclic triterpenoids, guiajaverin, and quercetin [41].

Flavonoids are also capable of preventing fat deposition and adipocyte differentiation due to high fat diet feeding in animals [42]. In this study guava leaf powder supplementation prevented the fat deposition in high fat diet fed rats. These findings were also supported by previous studies that showed that phenolic antioxidants decreased peritoneal fat deposition in diet induced obese rats. Recently we discussed the possible mechanism of increased fat metabolism in obesity by the application of citrus antioxidants and cinnamic acid derivatives [43, 44]. This study also provides evidence that guava leaf powder supplementation lowered the elevated level of cholesterol and triglyceride levels in HCHF diet fed rats. Increased cholesterol may also predispose hepatocyte dysfunction and the development of nonalcoholic fatty liver (NAFL) like diseases and steatosis [42]. Histological evidence also suggests that guava leaf powder supplementation ameliorated the fat deposition in liver of HCHF diet fed rats.

Oxidative stress is one of the early events that happen in tissues which ultimately develop metabolic syndrome in obesity [45]. Several mechanisms are responsible for reactive oxygen species (ROS) generation in obese individual which in turn triggers oxidative stress. Peroxisomal oxidation of fatty acids is the first procedure, which can produce ROS in oxidation reactions. The other mechanism is overconsumption of oxygen by mitochondrial respiratory chain which generates free radicals in oxidative phosphorylation [46, 47]. It is found that the concentration of lipid peroxidation product MDA was significantly elevated in liver of HCHF diet fed rats. This finding was also supported by our previous reports, suggesting that HCHF diet feeding increased the oxidative stress markers [25, 26]. Oxidative stress was further evidenced by the deposition of free iron in the tissues of HCHF diet fed rats. Tissue iron metabolism is a highly regulated process. Free iron is considered as the free radicle generator from water by Fenton like reaction. Previous studies suggested that iron deposition can be observed in various diseases in liver including nonalcoholic fatty liver diseases [48, 49]. Guava leaves powder supplementation prevented the deposition of iron deposition in liver of HCHF diet fed rats. This finding is also supported by our recent works showing that guava leaves powder can prevent iron deposition and oxydative stress in fluodrocortisone induced rats [50].

Oxidative stress mediated tissue damage also increases the liver markers enzyme activities. It mainly happens when there is any damage in the liver or deterioration in the liver starts, where damage to the hepatocytes increases the leakage of AST, ALT, and ALP into the blood stream. In the present study, HCHF diet induction caused a significant increase in the serum AST, ALT, and ALP activities in plasma compared to the control. Supplementation of guava leaves powder decreased these enzymes activities in plasma of HCHF diet fed rats. Hepatocyte damage due to oxidative stress and fat deposition also lead to inflammatory cells infiltration and fibrosis in the liver of HCHF diet fed rats. Our previous study showed that antioxidant rich food supplements may amelirate the inflammatory cells infiltration and fibrosis in HCHF diet fed rats [26]. Previous studies suggested that the activation of hepatic stellate cells due to increased oxidative stress is the main source of extracellular matrix protein collagen deposition [51]. Thus, preventaion of oxidative stress could be a valuable way of preventing fibrogenic response in tissues. In this study, guava leaves powder supplementation prevented the inflammation and decreased the collagen deposition in the liver of HCHF diet fed rats. Moreover, antioxidant enzymes like catalase and SOD activities in the liver of HCHF diet fed rats were seen to be significantly lower than those in control group which signifies that HCHF diet reduced the antioxidant capacity of the liver cells. These enzymes activities were increased and restored to near normal by guava leaf powder supplementation in HCHF diet fed rats, which indicates that guava leaf powder may improve the antioxidant capacity by increasing antioxidant enzymes.

## 5. Conclusion

In conclusion, the present study revealed that the supplementation of guava leaf powder prevents obesity and improves glucose intolerance, inflammation, and oxidative stress in liver of HCHF diet induced obese rats. This is only a primary initiative to establish guava leaf powder supplementation for obesity treatment; further studies such as clinical trials are required to develop it as one of the alternative treatments for obesity and obesity related metabolic syndrome.

## Figures and Tables

**Table 1:** Composition of normal and high carbohydrate high fat diet used in this study (for 100 g). Table 1 is reproduced from reference [25], [under the Creative Commons Attribution License/public domain].

**Ingredients of ** **Normal lab diet**	%	**Ingredients of HCHF diet**	%
Wheat	40%	Powdered normal rat feed	15.5%

Wheat Bran	20%	Sugar	17.5%

Rice Polishing	0.5%	Beef tallow(fat)	20.0%

Fish meal	1.0%	Condensed milk	39.5%

Oil cake	1.0%	Vit-B complex	0.1%

Gram	0.39%	Salt	0.5 %

Pulses	0.39%	Water	100 ml

Milk	0.38%		

Soybean Oil	0.15%		

Molasses	0.095%		

Salt	0.095%		

Vit-B complex	0.1%		

## Data Availability

The data used to support the findings of this study are included within the article.
